# Hemocompatibility of Inorganic Physical Vapor Deposition (PVD) Coatings on Thermoplastic Polyurethane Polymers

**DOI:** 10.3390/jfb3020283

**Published:** 2012-04-17

**Authors:** Juergen M. Lackner, Wolfgang Waldhauser, Paul Hartmann, Franz Bruckert, Marianne Weidenhaupt, Roman Major, Marek Sanak, Martin Wiesinger, Daniel Heim

**Affiliations:** 1 Joanneum Research Forschungsges.m.b.H., Institute for Surface Technologies and Photonics, Functional Surfaces, Leobner Straße 94, Niklasdorf A-8712, Austria; Email: Wolfgang.Waldhauser@joanneum.at (W.W.); Paul.Hartmann@joanneum.at (P.H.); 2 Grenoble Institute of Technology, Parvis Louis Néel, 38000 Grenoble Cedex 9, France; Email: Franz.Bruckert@grenoble-inp.fr (F.B.); Marianne.Weidenhaupt@phelma.grenoble-inp.fr (M.W.); 3 Institute of Metallurgy and Materials Sciences, Polish Academy of Sciences, Reymonta 25, Krakow 30-059, Poland; Email: nmrmajor@imim-pan.krakow.pl; 4 Department of Medicine, Jagiellonian University Medical College, Kopernika 23, Kraków 31-501, Poland; Email: msanak@cm-uj.krakow.pl; 5 Faculty of Technical and Environmental Sciences, University of Applied Sciences Upper Austria, Franz-Fritsch-Straße 11, Wels 4600, Austria; Email: martin.wiesinger@fh-wels.at (M.W.); Daniel.Heim@fh-wels.at (D.H.)

**Keywords:** inorganic thin film materials, diamond-like carbon, dynamic blood testing, blood

## Abstract

Biocompatibility improvements for blood contacting materials are of increasing interest for implanted devices and interventional tools. The current study focuses on inorganic (titanium, titanium nitride, titanium oxide) as well as diamond-like carbon (DLC) coating materials on polymer surfaces (thermoplastic polyurethane), deposited by magnetron sputtering und pulsed laser deposition at room temperature. DLC was used pure (a-C:H) as well as doped with silicon, titanium, and nitrogen + titanium (a-C:H:Si, a-C:H:Ti, a-C:H:N:Ti). *In-vitro* testing of the hemocompatibility requires mandatory dynamic test conditions to simulate *in-vivo* conditions, e.g., realized by a cone-and-plate analyzer. In such tests, titanium- and nitrogen-doped DLC and titanium nitride were found to be optimally anti-thrombotic and better than state-of-the-art polyurethane polymers. This is mainly due to the low tendency to platelet microparticle formation, a high content of remaining platelets in the whole blood after testing and low concentration of platelet activation and aggregation markers. Comparing this result to shear-flow induced cell motility tests with e.g., *Dictostelium discoideum* cell model organism reveals similar tendencies for the investigated materials.

## 1. Introduction

Biomedical devices (artificial hearts, lungs, kidneys, *etc.*) and interventional tools (stents, guidewires, catheters, *etc.*) play a major role in treating disease and organ insufficiency in the aging population. However, human immune reactions to implanted biomaterials work against the intention to heal disease, and may even cause serious harm or death [1]. When a biomaterial is implanted in a blood vessel in the human body, water and protein adsorption to surfaces immediately occurs. Then, cells such as leucocytes and platelets contact biomaterial surfaces and are activated due to stimulation by cytokines and growth factors [2,3]. Common failures of biomaterials arise from thrombogenesis. This thrombus formation occurs normally only at the site of vascular injury and involves platelet adhesion followed by the activation of blood clotting cascade and formation of fibrin clot. Thus, a significant factor determining hemocompatibility is the platelets’ response to the surface of a biomaterial, as indicated by their morphological changes [4,5]. Although blood platelets are a major morphotic component of thrombus, other cells, like erythrocytes and leukocytes, can also accumulate at the site of fibrin deposition. Activated leukocytes easily adhere to the surface of endothelial cells, and migrate out of the bloodstream into surrounding tissues.

Biomaterial improvements are generally achieved by surface modifications. Surface modification techniques either change the existing surface or apply an additional layer on the surface. Plasma techniques offer a great variety for such changes on metal, ceramics and polymer surfaces. Plasma sterilizes and cleans surfaces (from manufacturing residues), but also promotes adhesion for subsequent deposits (further coatings, proteins, cells) by chemical, mechanical and topographical changes. Basic processes are ablation to remove the outermost layer, implantation of oxygen or nitrogen atoms, radical formation on the surface, and deposition of polymers, metals or ceramic materials of generally a few nanometers to ~50 micrometers thickness. Many of the plasma-based deposition processes (physical and plasma-activated chemical vapor deposition) require elevated temperatures (>150–200 °C) to reach the desired properties of the surfaces. However, a lot of medically applied polymers can withstand only temperatures of up to 70–100 °C. Thus, there is increasing focus to develop low temperature physical and chemical vapor deposition techniques for deposition of e.g., inorganic coatings and diamond-like carbon (DLC).

Several studies reported about the biocompatibility of DLC coatings, like amorphous hydrogenated carbon (a-C:H) [6,7,8,9,10]. DLC generally consists of an amorphous matrix of sp2 and sp3 bonded carbon atoms. Carbon-based thin films with an increased fraction of sp3 bonds are known to possess high mechanical hardness, low surface roughness, chemical inertness, and exhibit good blood compatibility [11,12]. In addition, carbon layers developed onto polytetrafluoroethylene were observed to drastically increase adhesion and proliferation of human endothelial cells, which is beneficial for stent applications [13]. Further attempts to improve the anti-thrombus and physical properties have involved adding elements such as fluorine [1,14,15], silicon [16], phosphorus [17] and boron [18] to DLC. Fluorinated DLC films have performed particularly well in both blood adaptability and *in vivo* biocompatibility [15]. There are thought to be numerous mechanisms that prevent the adhesion and activation of platelets on DLC or doped-DLC, mediated proteins, chemical properties of the surfaces, wettability and surface structures. In contrast, increased binding of platelets was found for titanium and titanium nitride (TiN) surfaces and the state of platelet activation seemed to be more pronounced [19]. Comparative studies have also reported that DLC was found to be more hemocompatible than other biomaterials such as Ti, TiN, titanium carbide and carbonitrides [20,21,22,23]—however, with some controversial results.

The aim of this work is improving biomaterial surfaces by various metal and DLC based coatings, deposited at room temperature by magnetron sputtering and pulsed laser deposition (PLD) on thermoplastic polyurethane (PU) surfaces in order to achieve low platelet activation and aggregation.

## 2. Results and Discussion

The platelet’s main role in hemostasis is to preserve the integrity of the vascular wall through formation of a platelet plug. Testing the platelet function *in-vitro* in proximity to biomaterial surfaces requires dynamic testing to simulate the conditions resulting in platelet adhesion and aggregation, the most dangerous situation in blood contact. Platelets (PLT) respond to minimal stimulation—both during the transport in the flowing blood, which depends on the flow conditions, and the reaction with the surface, which depends on the nature of the surface and the adsorbed proteins [24].

PLT adhesion on (damaged) vessel walls occurs *in-vivo* under high shear forces, which are realized in this study *in-vitro* by the cone-and-plate (let) analyzer (CPA) setup. Primary PLT adhesion as the first step of primary hemostasis is characterized by initially reversible adhesion of still resting PLTs on the damaged vessel wall (or the biomaterial) [25], but can result in PLT activation (secondary adhesion) [26]. Activation occurs if adhering PLTs detect shear stresses that are too high (shear-induced pathway [27], or if they contact any thrombogenic surface such as injured endothelium, subendothelium and artificial surfaces (biomaterial induced PLT activation [28]).

**Figure 1:**
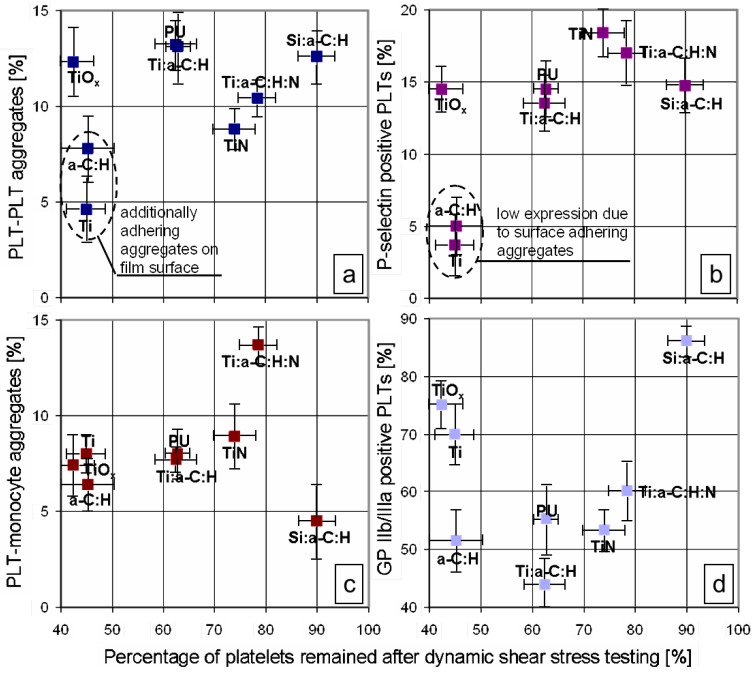
Plots ranking hemobiocompatibility of tested materials in dependency on the percentage of PLTs remaining after dynamic shear stress testing in the CPA device: (**a**) percentage of PLT aggregates; (**b**) percentage of P-selectin positive PLT; (**c**) percentage of PLT-monocyte aggregates; (**d**) percentage of GP IIb/IIIa positive PLTs.

PLT adhesion, activation and finally aggregation are regulated by glycoproteins (GP) at the PLT membrane, which recognize specific structural components of the extracellular matrix in the subendothelium and media. The GPs IIb-IIIa complexes play central roles and have the highest density on PLTs among the different PLT adhesion receptors (see e.g., [24]): In the shear-induced adhesion pathway [27], first the binding of von Willebrand factor (vWF) to the GP Ib receptor, then the expression of activated GP IIb-IIIa and release of PLT vWF, and finally, vWF binding to GP IIb-IIIa leads to irreversible adhesion. On resting PLTs, GP IIb-IIIa is in an inactive form and has a low-affinity binding site for adsorbed fibrinogen [29] and soluble plasma fibrinogen cannot bind to the PLT surface. Binding sites for fibrinogen in the region of GP IIb-IIIa only become accessible after PLT activation and conformational change. Thus, measuring PLTs with active GP IIb-IIIa complex gives detailed information about PLT function and fibrinogen binding: In our study (Figure 1d), GP IIb-IIIa was found to be evident in low concentrations in flow cytometry of the tested liquid for PU, a-C:H, a-C:H:Ti, TiN, and a-C:H:N:Ti, while a:C:H:Si, Ti, and TiO_x_ show significantly higher values. However, these results have to be discussed, including the adhering PLTs and PLT aggregates found in confocal microscopy: Such mechanisms occur for a-C:H and Ti, while contrariwise, no adhesion was found for all other analyzed biomaterials. This finding will become important in the further discussion as well.

While the initial binding of fibrinogen to GP IIb-IIIa is a reversible process (primary aggregation), it is followed seconds to minutes later by an irreversible stabilization of the fibrinogen linkage to the GP IIb-IIIa complex (secondary aggregation) [30]. In our study, CPA testing time was 5 min and about a similar amount of time was necessary subsequently for flow cytometry analyzing. Thus, we think that all GP IIb-IIIa positive PLTs are linked irreversibly to fibrinogen and have undergone the rearrangement of the PLT membrane and a drastic shape change [28]. Based on theories [31], the multivalent adhesion molecule fibrinogen possesses specific binding sites in the α- and γ-chains, which can bind to two GP IIb-IIIa molecules, whereby each is on a different PLT. Thus, PLT-PLT microaggregates are formed during aggregation. These microaggregates were analyzed for the biomaterial coatings and are presented in Figure 1a: On all non-PLT adhesive surfaces rather similar concentrations of PLT-PLT microaggregates are evident, only TiN has a significantly lower tendency to aggregation. Quite similar results were found for bigger aggregates >2–3 PLTs. The PLT adhesive surfaces Ti and a-C:H resulted in lower aggregate concentrations in the tested liquid.

PLT aggregation is accompanied by the release of Ca^2+^ and fibrinogen into the surrounding, stored in high concentrations in resting PLT granules. Two mechanisms of secretion (or degranulation) are known: (1) The granules can give up their contents to the extracellular space on coalescence with the open canicular system. (2) Direct secretion to the surrounding space can occur through coalescence of the granule’s membrane with the plasma membrane (exocytosis) [32]. Both granule secretion processes reinforce and boost the activation/agglomeration process, because the binding via the GP IIb-IIIa complex is strongly dependent on (high) Ca^2+^ concentrations. Reasonably, further circulating PLTs are recruited, and the PLT thrombus is consolidated by promoting the formation of fibrin [32]. 

Secretion of the α granules also results in the release of the glycoprotein P selectin from these granules, which is translocated to the membrane surface and to plasma [33]. P-selectin plays an important role in mediating adhesion of activated PLTs to neutrophils, monocytes and a subset of lymphocytes [34,35] and triggers inflammatory reactions in leukocytes. Flow cytometry analyses of P-selectin positive PLTs (Figure 1b) show no significant differences for all investigated materials (except for the PLT adherent Ti and a-C:H). Evaluation of the PLT-monocyte aggregates (Figure 1c) and PLT-neutrophile showed quite similar results for all materials as well, except for a-C:H:N:Ti and a-C:H:Si, being significantly higher and lower, respectively. Generally, the PLT-leukocyte aggregates are based on fibrinogen cross-linking via GP IIb-IIIa and Mac-1 on leukocyte (monocytes or neutrophils).

PLT activation and aggregation leads to a high consumption of PLTs, removing them from circulation. This is characterized by high PLT turnover rather than their adherence and by the formation of microemboli rather than occlusive thrombi formation. Following contact with the biomaterial (or the layer of adsorbed proteins on the artificial surface), PLTs will either adhere—as found for Ti and a-C:H—or “bounce off”, found for the other coatings and uncoated PU [36,37,38]. Considering this parameter in the ranking of the investigated biomaterial coatings leads to the abscissae in all diagrams in Figure 1andFigure 2. a-C:H:Si as well as a-C:H:Ti were found to be best materials regarding preventing PLT consumption. Overall, using this parameter for lining-up biomaterials in the diagrams defines the lower right part as most suitable for diminished interaction to PLTs and the upper left part as highly thrombogenic. Furthermore, it is not as simple to define a non-thrombogenic surface alone as “non-supporting PLT adhesion”. Moreover, this parameter of PLT consumption was proposed to be the most important in PLT hemocompatibility testing [39]. 

**Figure 2:**
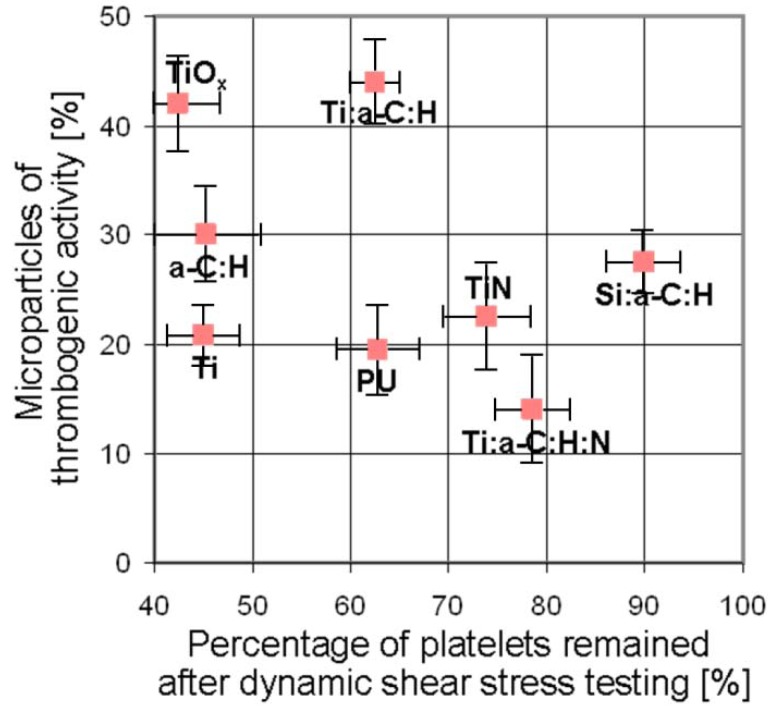
Concentration of microparticles with thrombogenic activity in dependency on the percentage of PLTs remained after dynamic shear stress testing.

Finally, PLT activation can result in the formation of PLT-rich microparticles (PMP) [33,40,41], which are particularly rich in factor Va, PLT factor 3 and phospholipid-like procoagulant activity (phosphatidylserine) [42,43]. Microparticle formation reflects fragmentation of PLTs and release of cell membrane vesicles during exocytosis and impose a thrombotic burden to circulating blood [39,40,41,42]. Recently, this was found to correlate with atherosclerosis and the risk for major adverse cardiovascular events, such as heart infarct or stroke [43,44]. Their detailed physiological role remains unclear, but *in-vitro* results have shown that they can bind and adhere to fibrinogen and fibrin, and coaggregate with PLTs [45,46]. Microparticles of thrombogenic activity were generally found to be increasing for decreasing PLT consumption. The best material in our studies regarding this aspect is a-C:H:N:Ti (Figure 2).

Finally, ranking of the investigated biomaterials coatings, concerning all the above-mentioned aspects, leads to a-C:H:N:Ti and TiN to be optimally applicable and much better than state-of-the-art PU, possessing the least thrombogenic surface and inducing least impact to the test blood. a-C:H:Si is a further candidate, however struggles with to high GP IIb-IIIa activation. High surface adhesion of PLTs, apparently improving the flow cytometry analysis of the liquid at first glance, and worst behavior regarding PLT consumption were found for Ti, a-C:H (and TiO_x_). 

Similar results of cell adhesion on these biomaterials were achieved by studying shear-flow-induced cell motility (SFICM) based on the setup used by Lauffenburger *et al.* [47,48,49,50] with *Dictyostelium discoideum* Ax-2 cell line model organisms (in their vegetative phase) (for details compare to [50]). The setup is based on a lower horizontal disc (titanium or silicon with the different biomaterial coatings) and a second, upper disc (stainless steel) with a small centre hole in 0.1 mm distance (see for details [51]). *D. discoideum* cells are initially placed on the lower discs and washed away during testing if the exerted shear forces by Sörensen phosphate buffer introduced by the centre hole are too high. Such conditions are found close to the centre hole—the critical shear stress is defined for the position, where 50% of cells are detached under time-dependent equilibrium conditions. In this study, extremely high hydrodynamic stress (>60 Pa) is required to detach *D. discoideum* cells from a-C:H surfaces independently on the substrate. Slightly lower threshold stress was found for Ti (~40 Pa). Lowest adhesion is found for TiN and a-C:H:N:Ti (10–20 Pa) [51]. Although *D. discoideum* cells and PLTs are limitedly comparable, the similarity in results is astonishing—even the stress level for attachment is comparable: In the CPA device we used bet 10–15 Pa shear stress for PLT function testing (*in-vivo* in healthy vessels 1–2 Pa and increase to 10–15 Pa during vessel injury).

The origin of the differences in the biomaterial behavior seems to be the protein adsorption: During first contact with blood, a biomaterial surface is mainly coated with albumin, immunoglobulins and fibrinogen from plasma. Adhesion of PLTs to fibrinogen onto these surfaces is not as irreversible as to collagen coated with vWF multimers of a damaged blood vessel [52]. Activation by adenosine diphosphate (ADP) is required for resting PLTs to efficiently adhere to fibrinogen [53]. Furthermore, fibrinogen appears to mediate PLT aggregation efficiently only under low shear stress conditions (1–3 Pa), but not at high shear stress (>5 Pa) [54], which occur e.g., in damaged vessels and were used in our tests. Our studies of bovine serum albumin (BSA) and fibronectin (Fn) adsorption, presented in detail elsewhere, revealed lower BSA adsorption for Ti and TiO_x_. However, it is even much higher than found for PU. All other coatings have quite similar adsorption of BSA. Fn adsorption is also similar for all coatings, but about 5 times higher than for uncoated PU. Such behavior can be the explanation for worse PLT function by high PLT consumption and high concentration of GP IIb-IIIa positive PLTs of these materials. Surface charge could be another influence on the protein adsorption properties, which will be applied in future research. 

## 3. Experimental Section

### 3.1. Substrate Materials, Surface Modification and Deposited Films

In the current work, we used Pulsed Laser Deposition (PLD) for ablating the metal target surface by a high power, pulsed laser beam (Nd:YAG laser, Powerlite/Continuum Inc/Santa Clara/CA/US, 1064 nm wavelength, ~10 ns pulse duration, 50 Hz pulsing rate) [55,56] after evacuation of the chamber to pressures <2 × 10^3^ Pa and a pretreatment (activation) step by a linear anode layer ion beam (Veeco Inc./Fort Collins/CO/US) in argon-oxygen plasma [57]. Additionally, coatings were deposited after similar evacuation and pretreatment by magnetron sputtering with argon sputter gas and direct current (DC), high frequency (HF = pulsed DC) and radio frequency (RF) power. The substrate temperature in the industrially-scaled, large-area and high rate PLD and magnetron sputtering equipment (Pfeiffer Vakuum/Vienna/Austria, Leybold Vakuum/Cologne/Germany) was kept at room temperature (max. 25 °C). Several different film materials were deposited by these techniques: pure titanium (Ti), titanium nitride (TiN), titanium oxide (TiO_x_), a-C:H, silicon, and titanium doped a-C:H (a-C:H:Si, a-C:H:Ti) as well as titanium plus nitrogen doped a-C:H (a-C:H:N:Ti). Materials and deposition techniques are listed in Table 1. Gases were supplied by Linde Gas GmbH (Stadl-Paura/Austria) in 99.996% purity for Ar, N_2_ and O_2_ and 99.5% purity for acetylene (C_2_H_2_). 

**Table 1:** Film materials and deposition parameters.

Material	Deposition technique	Target material	Target manufacturer	Process gases
Ti	Sputter (DC)	Ti (grade 2)	Eurotitan GmbH, Solingen, Germany	Ar
TiN	PLD	Ti (grade 2)		N_2_, Ar
TiO_x_	Sputter (HF)	Ti (grade 2)		O_2_, Ar
a-C:H	Sputter (HF)	Electrocarbon graphite (99.7% carbon)	Hoffmann & Co., Steeg, Austria	C_2_H_2_, Ar
Si:a-C:H	Sputter (RF)	Ti (grade 2)	Eurotitan GmbH, Solingen, Germany	C_2_H_2_, Ar
Ti:a-C:H	Sputter (HF)	Ti (grade 2)		C_2_H_2_, Ar
Ti:a-C:H:N	PLD	Ti (grade 2)		C_2_H_2_, N_2_, Ar

**Figure 3:**
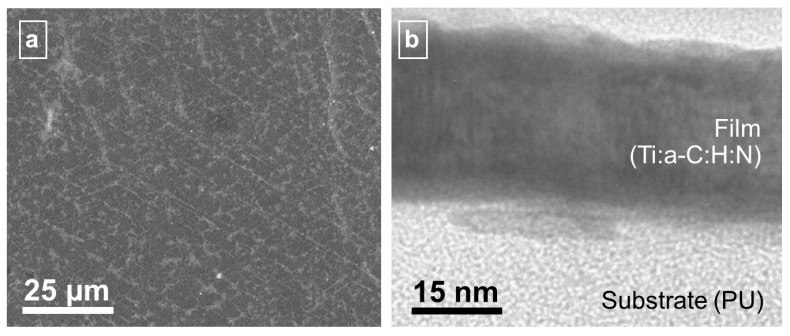
(**a**) Surface topography (analyzed by scanning electron microscopy) and (**b**) growth structure (analyzed by high resolution transmission electron microscopy) of typical Ti:a-C:H:N films on PU substrate.

The deposition resulted in 20 nm thick films on 2 mm thick thermoplastic polyurethane substrates (PU, Elasthane 55D, DSM PTG/Berkeley/CA/US, thermal stability ~65°), kindly provided by the Foundation for Cardiac Surgery Development/Zabze/Poland. Additionally, 300 nm thick films were grown on silicon wafers (B-doped, Wacker Chemie AG/München/Germany) and Grade-2 titanium (Eurotitan GmbH/Solingen/Germany) substrates. Silicon wafers had 2" diameter and 500 µm thickness, titanium discs 2 mm thickness. Ti and stoichiometric TiN films were found to be nanocrystalline, all other films amorphous (see cross section in Figure 3b) with strong binding to the PU substrate (no delaminations). Si:a-C:H films contain 8.4% Si, Ti:a-C:H films ~10% Ti. Ti:a-C:H:N films contain ~19% Ti and ~10% N (for details including chemical binding and hydrogen contents in DLC films compare [58]). Hydrogen contents were found to be ~20–35 at. % of the diamond-like carbon coatings.

The microtopography of the substrate materials is not altered by the coating (see SEM image in Figure 3a). The nanotopography on PU is roughened by stress-induced formation of ~5 nm high hills, separated 15 nm from one another (for details see [59]). The surface energy of the coatings on Si substrates in the same state as prepared for hemocompatible testing was measured based on sessile contact angle measurements with distilled water, diiodo methane and ethylene glycol 50% water (Carl Roth/Karlsruhe/Germany) and found rather similar range with polar contributions of 35–44 mN/m and disperse contribution of 5–12 mN/m. TiO_x_ and TiN as well as Ti and a-C:H:N:Ti are on the lower level of this energy range (below 40 and 45 mN/m, respectively), while all other coating types are at the upper limit. Bacterial testing of the coatings with Eschierichia coli XL-blue (Agilent Technologies Inc./Loveland/CO/USA) revealed improved behavior of a-C:H, Ti, TiN and a-C:H:Ti compared to uncoated PU in both optical inspection of 12 h/37°C cultivated bacteria coated samples as well as bacterial counting in LB and Agar medium.

### 3.2. Platelet Function Testing

#### 3.2.1. Materials

A fasting venous blood (4 × 4.5 mL) was drawn from the same male healthy donor (age 50 years) into sodium citrate tubes (0.5 mL, 0.105M concentration, BD Vacutainer Systems, Franklin Lakes, NY, USA) at one week intervals. A wide gauge needle (12 G) was used for blood sampling without any stasis to prevent platelet activation. For the same reason, the first sampling tube was rejected. Measurement of complete blood count was done using Sysmex K-1000 cell counter (Sysmex Co., Mundelein, IL, USA) calibrated each day. Aliquots of blood (0.4 mL) were stored at room temperature and were used for testing within 45 min. An additional blood aliquot was sampled at the time of the shear stress to investigate impact of static storage on platelet function. Another aliquot of blood was activated with adenosine diphosphate (ADP, 20 μM final concentration) for 5 min. The control samples were processed in parallel with each replicate of the shear stress experiment. These samples served baseline control for the static conditions and to test platelets capacity to activate.

#### 3.2.2. Cone-and-Plate(let) Analyzer Testing

Testing of materials in the shear stress conditions was performed with a CPA device (Impact-R, DiaMed AG, Cressier (FR), Switzerland). The CPA is a commonly used instrument, validated for clinical use in the evaluation of thrombotic diseases and the effectiveness of anti-platelet drugs. It mimics vascular flow and—in the used modified setup—dynamic interaction between whole blood and biomaterial [60,61]. The rationales for using this test were: (1) lower requirements of donor blood to perform the test, (2) availability of commercial testing instrument, (3) accepted principle of a rotating cone and plate as a source of shear stress, as it is used in rheometers or viscometers [62].

**Figure 4:**
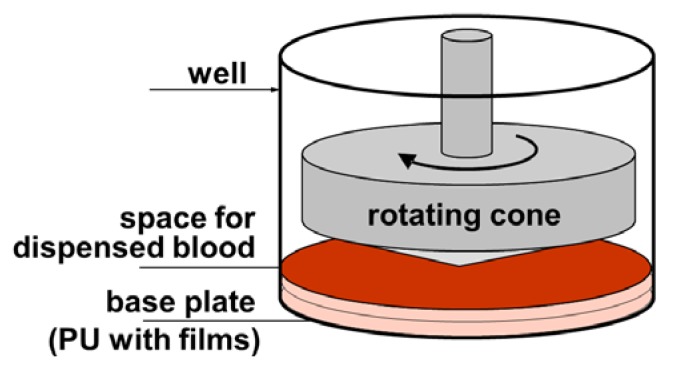
Schematics of the cone-and-platelet analyzer (CPA) setup including the base plate, consisting of PU and biomaterial films deposited thereon.

As recommended by the manufacturer, 130 μL blood volume was used for each shear stress test and the aliquot of the blood was gently mixed for 60 seconds on the rotational wheel (10 revolutions per minute) to prevent sedimentation of blood cells before each of the replicates. Polystyrene (PS) being the standard disposable insert well of the Impact-R kit was used as reference for testing platelets and for test validation. For the biomaterial coating testing, these inserts were exchanged by the specimen (coated polyurethane), which were cut to disks of 19.5 mm diameter and 2 mm thickness, which fitted the insert well tightly (see Figure 4).

The shear test was applied at a shear rate 1,800 s^−1^ (720 revolutions per minute) for 300 seconds, using a disposable PTFE conical rotor. Following the shear test, the rotor was carefully removed and blood was immediately sampled from the well to the test tubes for flow cytometry staining. From the remaining blood (80 μL), plasma was separated by centrifugation at 2,000 g for 5 min and stored frozen in −70 °C for further analysis of thrombotic activity.

#### 3.2.3. Flow Cytometry for Platelet Analysis in Blood

Two sets of tubes were prepared for each tested plate and for the static controls. Expression of platelet activation markers was ascertained using whole blood staining. In brief, 5 μL aliquots of blood were gently mixed with fluorochrome-conjugated monoclonal antibodies: 5 μL FITC-PAC-1, 5 μL PE-CD62P and 4 μL PerCP-CD61 (all from Becton Dickinson, USA) in phosphate buffered saline (PBS) containing 0.2% bovine serum albumin and 2 mM calcium chloride (final volume 35 μL). After 10 min staining at room temperature, erythrocytes were lysed by addition of 0.5 mL lysing solution (FLS, Becton Dickinson, Franklin Lakes, NJ, USA) and platelets were centrifuged (1,000 g, 6 min) and resuspended in PBS buffer for further analysis by flow cytometry. Samples were analyzed using EPICS XL flow cytometer (Beckman Coulter Inc., Brea, CA, USA). Expression of platelet activation markers [42] was measured on CD61 gated objects using PAC-1 antibody for conformational change of glycoprotein IIb/IIIa, and using CD62P for P-selectin. Integrated fluorescence of the activation marker was calculated as a multiplication total of geometric mean fluorescence by percentage of marker-positive objects. Aggregates of platelets were analyzed after erythrocyte lysis by mixing 25 μL of blood with 0.4 mL FLS and subsequent fixation by the addition of 3.5 mL 1% paraformaldehyde in PBS. Cellular material was recovered by centrifugation (1,000 g, 7 min) and immunostained (25 μL aliquots) with 4 μL PerCP-CD14 and 5 μL FITC-CD61 or 5 μL FITC-CD61 alone for 30 min at room temperature. Samples were then washed in PBS, and analyzed by flow cytometry. The percentage of granulocyte-platelet aggregates (leukocytes stained with platelet marker CD61) was calculated using granulocyte forward/side scatter gate and additional monocyte CD14+ gate. The absolute number of platelets was calculated as the number of CD61 positive objects in reference to total granulocytes count. Small and big platelet aggregates were counted using forward/side scatter gates for CD61 positive objects. All other chemicals were obtained from Sigma-Aldrich.

#### 3.2.4. Thromogenic Microparticle Activity Analysis

Thrombogenic potential of blood plasma was measured using ZymuphenMP-activity ELISA kit (Hyphen Biomed, Eragny, France), according to the manufacturer’s instructions. This assay is based on trapping phospholipid-rich microparticles derived from cell membranes, using immobilized annexin V, followed by reconstitution of thrombin activity with calibrated clotting factors solution. Proteolytic activity of generated thrombin against the chromogenic substrate strictly correlates with concentration of microparticles present in blood plasma [63].

### 3.3. Statistics

Statistical analysis was performed using ORIGIN Pro version 7.5 G and SPSS version 17.0 statistical packages. Data are presented as means ± SD (standard deviation) and ranges. Statistical significance was assumed for type-I error p < 0.05. 

## 4. Conclusions

Magnetron sputtering as well as pulsed laser deposition was used to deposit titanium-based inorganic thin film materials (Ti, TiO_x_, TiN) as well as pure and doped DLC coatings of ~20 nm thickness on thermoplastic polyurethane surfaces. *In-vitro* testing the hemocompatibility under dynamic test conditions in an adapted device based on a cone-and-plate analyzer revealed for Ti, TiN and nitrogen doped DLC optimal anti-thrombotic behavior, being better as for the state-of-the-art polyurethane polymers (gold standard in clinics). This is due to the low tendency of platelet activation, aggregation and microparticle formation. 
